# *Francisella tularensis* D-Ala D-Ala Carboxypeptidase DacD Is Involved in Intracellular Replication and It Is Necessary for Bacterial Cell Wall Integrity

**DOI:** 10.3389/fcimb.2018.00111

**Published:** 2018-04-10

**Authors:** Petra Spidlova, Pavla Stojkova, Vera Dankova, Iva Senitkova, Marina Santic, Dominik Pinkas, Vlada Philimonenko, Jiri Stulik

**Affiliations:** ^1^Department of Molecular Pathology and Biology, Faculty of Military Health Sciences, University of Defence, Hradec Kralove, Czechia; ^2^Department of Microbiology and Parasitology, Medical Faculty, University of Rijeka, Rijeka, Croatia; ^3^Microscopy Center, Institute of Molecular Genetics ASCR v.v.i., Electron Microscopy Core Facility, Prague, Czechia; ^4^Department of Biology of the Cell Nucleus, Institute of Molecular Genetics ASCR v.v.i., Prague, Czechia

**Keywords:** *Francisella*, DacD, virulence, phagosomal escape, carboxypeptidase, penicillin binding proteins, membrane defects

## Abstract

D-alanyl-D-alanine carboxypeptidase, product of *dacD* gene in *Francisella*, belongs to penicillin binding proteins (PBPs) and is involved in remodeling of newly synthetized peptidoglycan. In *E. coli*, PBPs are synthetized in various growth phases and they are able to substitute each other to a certain extent. The DacD protein was found to be accumulated in fraction enriched in membrane proteins from severely attenuated *dsbA* deletion mutant strain. It has been presumed that the DsbA is not a virulence factor by itself but that its substrates, whose correct folding and topology are dependent on the DsbA oxidoreductase and/or isomerase activities, are the primary virulence factors. Here we demonstrate that *Francisella* DacD is required for intracellular replication and virulence in mice. The *dacD* insertion mutant strain showed higher sensitivity to acidic pH, high temperature and high osmolarity when compared to the wild-type. Eventually, transmission electron microscopy revealed differences in mutant bacteria in both the size and defects in outer membrane underlying its SDS and serum sensitivity. Taken together these results suggest DacD plays an important role in *Francisella* pathogenicity.

## Introduction

Bacteria possess many tools that enable them to resist unfriendly environments, such as hostile intracellular milieu and host immune response, in order to survive the effects of antimicrobial agents and, inter alia, changing ion concentration. Their cell wall and its structure protect them against these negative influences. However, these surface structures, such as lipopolysaccharide and peptidoglycan, are triggers for a host cell's primary immune response (Chandler and Ernst, 2017).

One key component of the bacterial cell wall is the middle segment called peptidoglycan layer, which is connecting link between outer and inner membrane in gram-negative bacteria. Peptidoglycan macromolecule, i.e., sacculus, consists of individual glycan chains crosslinked by short peptides (Egan et al., 2015). Peptidoglycan sacculus is responsible for maintaining bacterial shape as well as providing mechanical strength to resist osmotic challenges (Vollmer et al., 2008a). The synthesis of peptidoglycan is divided into three steps with glycosyltransferases play an important role by polymerizing the glycan chains and DD-transpeptidases, that crosslink the peptides. Delivery of new material to the peptidoglycan layer occurs during transpeptidation. Because of this, there is need for some cleavage mechanisms to disrupt older layer and thus maintain the thickness of peptidoglycan. Cleavage is also required for cell division. This mechanism is ensured by peptidoglycan hydrolases (Vollmer et al., 2008b). Newly synthesized peptidoglycan is rich on pentapeptides, which are reduced to tetrapeptides by DD-carboxypeptidases. Some tetrapeptides are trimmed to tripeptides by LD-carboxypeptidases (Glauner and Höltje, 1990).

Penicillin binding proteins (PBPs) are components of peptidoglycan biosynthesis (Matsuhashi, 1994). In *E. coli* 12 PBPs were identified. The first seven were named in order of their decreasing molecular weight (PBP 1a, 1b, 2, 3, 4, 5, 6; Spratt, 1975). The remaining five PBPs were described later: PBP 7 (Henderson et al., 1994), PBP 8 (Henderson et al., 1995), DacD (Baquero et al., 1996), AmpC and AmpH (Henderson et al., 1997), PBP 1c (Schiffer and Höltje, 1999). Generally, PBPs are divided to the two main categories of either (HMW) or low molecular weight (LMW). HMW's are involved in the synthesis of peptidoglycan and its incorporation into the sacculus, namely PBPs 1a, 1b, 1c, 2, and 3. LMW's *in vitro* biochemical capabilities have been already determined but *in vivo* functions are still remain unclear (Denome et al., 1999). Members of this class are PBPs 4, 5, 6, and 7, DacD, AmpC, and AmpH (Nelson and Young, 2001). PBPs are expressed in various cell growth phases and can substitute each other to a certain extent (Ghosh et al., 2008).

Our subject of interest is one of LWM PBPs with DD-carboxypeptidase activity DacD, product of *dac*D gene in *Francisella* (locus *FTS_1034* in FSC200 strain). In *E. coli* DacD, often denoted as PBP6b, is expressed in stationary phase (Baquero et al., 1996). Its amino acid sequence homology (48%) with PBP5 suggests its role in β-lactam resistance, but deletion of *dacD* did not change the β-lactam resistance (Sarkar et al., 2011). Some studies have shown its role in biofilm formation in *Salmonella enterica* and *E. coli* (Brambilla et al., 2014). Recently a study was published that indicated *E. coli* PBP6b as being most active and most abundant at a low pH (pH 5), which suggests its necessity for growth and maintenance of bacterial cell shape in an acidic environment (Peters et al., 2016).

The relationship between DacD and acidic pH resistance could be interesting in connection with intracellular pathogen such as *Francisella*, the causative agent of tularemia. It is assumed that this gram-negative bacterium is able to resist the acid environment in the phagosome, escape to the cytosol of host cell, replicate there, cause host cell death and re-infect next host cells (Celli and Zahrt, 2013). Here, we show that the mutant strain with inactivated gene coding for DD-carboxypeptidase DacD is unable to replicate inside host as effectively as the wild-type counterpart although it escapes more rapidly from phagosomes. Furthermore, the mutant bacteria are more sensitive to several stress stimuli and also demonstrate the defects in the membrane integrity and the cell shape. These results document the role DacD in *Francisella* virulence contributing to the understanding of the mechanisms behind *Francisella* pathogenesis.

## Materials and methods

### Bacterial strains, plasmids, and growth conditions

Bacterial strains and plasmids used in this study are listed in Table 1. All work with *F. tularensis* subsp. *holarctica* FSC200 and derivative strains were conducted under BSL-2 containment. *Francisella* strains were cultured on McLeod agar plate enriched for bovine hemoglobin (Becton Dickinson, Cockeysville, MD, USA) and IsoVitalex (Becton Dickinson, Cockeysville, MD, USA) or grew in Chamberlain medium (Chamberlain, 1965) with shaking 200 rpm at 37°C. The *E. coli* strains were cultured in Luria Bertani (LB) broth medium or on LB agar plates. When appropriate, antibiotics were used at following concentration: kanamycin 20 μg/ml (*F. tularensis*) or 50 μg/ml (*E. coli*) (kanamycin sulfate, Serva, Heidelberg, Germany), ampicillin 100 μg/ml (*E. coli*) (ampicillin Na-salt, Serva, Heidelberg, Germany).

**Table 1:** Bacterial strains and plasmids used in this study.

**Strain**	**Description**	**Source**
*Francisella tularensis* FSC200	*Francisella tularensis* subsp. *holarctica*, wild-type, clinical isolate	*Francisella* Strain Collection (FSC) of the Swedish Defense Research Agency, Umeä, Sweden
FSC200/*in dacD*	*dacD (FTS_1034)* insertion mutant strain	This study
FSC200/*dacDtrans*	Insertion mutant strain complemented *in trans*	This study
FSC200/*dacDcis*	Insertion mutant strain complemented *in cis*	This study
*E. coli* S17-1 λpir	*Escherichia coli* donor strain for conjugation TpR SmR recA, thi, pro, hsdR-M+RP4: 2-Tc:Mu: Km Tn7 λpir	Simon et al., 1983
*E. coli* XL1	*Escherichia coli* competent cell *recA1 endA1 gyrA96 thi-1 hsdR17 supE44 relA1 lac* [F′ *proAB lacIq Z*ΔM15 Tn*10* (Tet^R^)]	Stratagene
**Plasmid**	**Description**	**Source**
pCR4-TOPO	Cloning vector, pUC *ori*, P*_*lac*_, lacZ*, Kan^R^, Amp^R^	Invitrogen
pDM4	*F. tularensis* suicide vector, *mob*_RP4_,*ori*_R6K_, *sacB*, Cm^R^	Milton et al., 1996
pKK289KmGFP	*E. coli/ F. tularensis* shuttle vector, Ft *ori*, p15a *ori*, Km^R^, groES promoter	Bönquist et al., 2008

All chemicals without the specified manufacturer were purchased from Sigma-Aldrich (St. Louis, MO, USA).

### TargeTron insertional mutagenesis

The TargeTron gene knockout system was used to generate the *dacD* mutant (Rodriguez et al., 2008) (Sigma-Aldrich, St. Louis, MO, USA). The primers used in plasmid construction are listed in Table S1. The PCR product was digested (*Hind*III-*Bsr*GI, NEB, Ipswich, MA, USA) then inserted into the *Francisella* targeting vector pKEK1140 (generously provided by Karl Klose, University of Texas at San Antonio, San Antonio, TX) to generate a TargetTron insertion plasmid (Rodriguez et al., 2008). Constructed plasmid DNA was introduced into the FSC200 strain by electroporation. The presence of the TargeTron insertion was determined by using an intron-specific EBS universal primer combined with a gene specific primer. Intron insertion of the targeted gene was determined by using gene-specific primers that amplified across the insertion site. Selected positive clones grew in Chamberlain medium at 37°C overnight then plated on McLeod agar plate (without kanamycin) and incubated at 37°C to remove the TargeTron temperature—sensitive plasmid. The insertion mutants were confirmed by using PCR with the gene-specific primers and denoted FSC200/*in dacD*.

### Functional complementation

The functional complementation in generating the *dacD* complemented strain included complementation *in trans* and *in cis*. A DNA fragment carrying the wild-type *dacD* gene was PCR amplified by using FSC200 genomic DNA as a template, employing primers DacD_pKK289_F and DacD_pKK289_R (Table S1). The final PCR product was sequenced, then cloned downstream of the GroES promoter by replacing the green fluorescence protein-encoding gene in the shuttle vector pKK289gfp (Bönquist et al., 2008). The constructed plasmid DNA pKK289dacD was introduced into the mutant strain FSC200/*in dacD* by electroporation. The strain complemented *in trans* was denoted FSC200/*dacDtrans*. For complementation *in cis* the *dacD* gene with flanking regions was amplified by using primers DacDcis_F and DacDcis_R. The PCR product was inserted into pCR4.0 TOPO TA cloning vector (Invitrogen, Carlsbad, CA, USA) to facilitate sequencing (Institute of Microbiology, Prague, Czech Republic). Using *Xho*I/*Xba*I the fragment was cut out, and then inserted into pDM4 suicide vector (Milton et al., 1996) linearized by the same restriction endonucleases digestion (NEB, Ipswich, MA, USA) to generate the *dacDcis* plasmid. Conjugal mating between *E. coli* S17-1 λpir donor strain (Simon et al., 1983) and *F. tularensis* FSC200/*in dacD* mutant strain followed by sucrose-selection led to the allelic exchange on the mutant strain chromosome and the resulting strain was denoted FSC200/*dacDcis*.

### Isolation of macrophages and *in vitro* proliferation

Mouse bone marrow cells were harvested from 6 to 10 weeks old, female BALB/c mice femur (Velaz, Czech Republic). The cavity of the femur was flushed out with DMEM (Invitrogen, Carlsbad, CA, USA) and bone marrow cells were collected. Washing bone marrow cells with pre-warmed DMEM two times then the cells were resuspended in BMMs medium [DMEM supplemented with 10% fetal bovine serum (FBS, Dominique Dutscher, Brumath, France) and 10% L929-conditioned medium as a source of macrophage colony stimulating factor] with appropriate antibiotics (50 U/ml penicillin, 50 μg/ml streptomycin; Sigma-Aldrich, St. Louis, MO, USA). The cells were incubated at concentration of 6 × 10^6^ cells per Petri dish for 1 week to differentiate into bone marrow-derived macrophages (BMMs). The day before infection, macrophages were seeded on tissue culture plates at the concentration of 5 × 10^5^ cells per well in antibiotic-free DMEM supplemented with 10% fetal bovine serum. Following cultivation overnight, BMMs were infected with all four bacterial strains (FSC200, FSC200/*in dacD*, FSC200/*dacDtrans*, and FSC200*/dacDcis*) at a multiplicity of infection (MOI) of 50. Actual infection doses were determined by plating serial dilutions of the culture inoculum. Infection started by centrifugation of plates for 5 min, 400 × g, and then the samples were incubated at 37°C, 5% CO_2_ for 30 min. Extracellular bacteria were killed during incubation in DMEM with 5 μg/ml gentamicin (Sigma-Aldrich, St. Louis, MO, USA) at 37°C, 5% CO_2_ for 30 min. The cells were washed three times with pre-warmed PBS before adding the DMEM with 10% FBS without antibiotics. At set time points (1, 6, 24, and 48 h), infected cells were lysed by 0.1% sodium deoxycholate, and then the lysates were plated on McLeod agar plates in a serial dilution. The plates were incubated at 37°C, 5% CO_2_ for 5 days. The number of viable intracellular bacteria was determined by colony forming units (CFU) counting.

Type II pulmonary epithelial cell line A549 (ATCC® CCL-185™) was cultured in DMEM supplemented with 10% FBS at 37°C, 5% CO_2._ Cell were seeded at a concentration of 1.5 × 10^5^ cells/well and let to adhere overnight. Cells were infected at a MOI of 100. Infection started by centrifugation of plates for 5 min, 400 × g, and then the samples were incubated at 37°C, 5% CO_2_ for 2 h. Extracellular bacteria were killed during incubation in DMEM with 25 μg/ml gentamicin for 2 h at 37°C, 5% CO_2_. The cells were washed three times with pre-warmed PBS before adding the DMEM with 10% FBS without antibiotics. At set time points (6, 24 and 48 h), the infected cells were lysed by 0.1% sodium deoxycholate, then the lysates were plated on McLeod agar plates in a serial dilution. The plates were incubated at 37°C, 5% CO_2_ for 5 days. The number of viable intracellular bacteria was determined by colony forming units (CFU) counting.

### Macrophage cytotoxicity assay

For cytotoxicity experiments, BMMs were seeded in 96-well tissue culture plates at a concentration of 2 × 10^4^ cells/well and allowed to adhere overnight at 37°C, 5% CO_2_. The next day, the BMMs were infected with bacterial cell suspensions at an MOI of 50:1. Following 30 min incubation, the extracellular bacteria were killed by gentamicin (5 μg/ml, 30 min), which corresponds to time zero. At 0, 24, and 48 h postinfection, culture plates were centrifuged to pellet cells (300 × g, 3 min) and the supernatant was collected. The activity of lactate dehydrogenase (LDH) in the supernatant was measured according to manufacturer's instructions (PIERCE LDH Cytotoxicity Assay kit, Thermo Fisher Scientific, Waltham, MA, USA) as an absorbance at the wavelength of 490 nm using the Paradigm microplate reader (Beckman Coulter, Brea, CA, USA). As a positive control (representing 100% cell lysis), uninfected BMMs were lysed with 0.1% sodium deoxycholate. Sample absorbance values were expressed as a percentage of the positive-control value.

### Infection of mice

Groups of five, 6–10 weeks old female BALB/c mice (Velaz, Czech Republic) were challenged by subcutaneous route at the dose of 10 and 50 CFU/mouse for FSC200/*in dacD* mutant; 10 CFU/mouse for FSC200, complemented strains FSC200/*dacDtrans* and FSC200*/dacDcis*. Control group of mice was challenged with physiological saline solution. Mice were observed for 21 days for morbidity and mortality.

For bacterial dissemination study, mice were challenged by subcutaneous route at a dose of 10 CFU/mice for each tested strain. At set time points, 3 BALB/c mice were euthanized by carbon dioxide exposure. The liver, spleen, and lung were processed for plating to determine the presence of bacteria.

### Standard and stress growth kinetics

*F. tularensis* strains were grown overnight at 37°C in Chamberlain medium supplemented with kanamycin (20 μg/ml) when applicable. The cultures were diluted with fresh Chamberlain medium to OD_600_ = 0.1. 200 μl aliquots of the diluted culture were transferred into a 96-well plate in pentaplicates with either the following stress conditions: pH 4.0, 3% NaCl, or 20 μM CuCl_2_, then samples were incubated at 37°C for 24 h. For heat stress study, samples were incubated at 42°C for 24 h. The growth kinetics was determined by measuring the OD_600_ every 10 min using a microplate reader FLUOstar Optima (BMG Labtech, Germany). Experiment was repeated three times.

### SDS sensitivity assay

*F. tularensis* strains were grown overnight at 37°C in Chamberlain medium, when required the kanamycin (20 μg/ml) was added. The bacterial cultures were diluted with 5 ml of fresh Chamberlain medium to get a bacteria working solution at final concentration equal to 10^8^ bacteria/ml and SDS to a final concentration of 0.05% was added. The number of viable bacteria was determined by plating a serial dilution of bacterial cultures on McLeod plates at set time points (0, 1, 2, 3, and 4 h after SDS addition). Bacteria were enumerated after 72 h incubation at 37°C. Experiments were repeated independently three times and data represent the average of all experiments.

### Transmission electron microscopy

BMMs were seeded in 24-well tissue culture plate at a concentration of 2 × 10^5^ cells/well and allowed to adhere overnight at 37°C, 5% CO_2_. Next day, BMMs were infected with bacterial cell suspensions at a MOI of 50. At appropriate time intervals (10 min, 30 min, 1 and 6 h) BMMs were fixed with 3.8% paraformaldehyde for 30 min at RT and then neutralized with 50 mM NH_4_Cl for 10 min at RT. Cells were quickly washed with Sörensen buffer (0.1 M sodium/potassium phosphate buffer, pH 7.3; SB) at 37°C, fixed with 2.5% glutaraldehyde in SB for 2 h, washed with SB, and postfixed with 1% OsO_4_ solution in SB for 2 h. The cells were dehydrated in series of ethanol with increasing concentration, subsequently in propylene oxide, and embedded in mixture of Epon 812 substitute and Durcupan ACM (Sigma-Aldrich, St. Louis, MO, USA).

Bacteria cultured in Chamberlain medium were fixed with 2.5% glutaraldehyde in SB for 1 h, washed with SB, resuspended in small volume of SB and mixed with 2% agarose in 1:1 ratio at 37°C. After centrifugation, the pellet was chilled, cut, washed with SB and postfixed with 1% OsO_4_ solution in SB for 2 h. The bacteria were dehydrated in series of ethanol with increasing concentration, subsequently in propylene oxide, and embedded in mixture of Epon 812 substitute and Durcupan ACM.

Polymerized blocks were cut into 80 nm ultrathin sections, collected on 200 mesh size copper grids, and stained with saturated aqueous solution of uranyl acetate for 4 min. The sections were examined in FEI Morgagni 268 transmission electron microscope (FEI, Eindhoven, The Netherlands) operated at 80 kV. The images were captured using Mega View III CCD camera (Olympus Soft Imaging Solutions, Münster, Germany).

For quantification of cell size differences, multiple random TEM images of wild-type and mutant cells taken at equal magnification of 11,000 x were used. Cell profiles were segmented using machine learning algorithms in ilastik software (Sommer et al., 2011), and areas determined in Fiji ImageJ (Schindelin et al., 2012). Total of 1,945 wild-type and 173 mutant cells were analyzed. Cell profiles exhibiting membrane defects were counted on the same images.

### Serum sensitivity assay

Bactericidal assay was conducted with fresh human serum prepared from the whole blood of the anonymous healthy non-immune donors. Briefly, collected nonheparinized whole blood from donors was kept at room temperature for 1 h, then stored at 4°C for 30 min to allow blood to clot. The blood clot was removed by centrifugation at 500 × g for 30 min at 4°C. The serum fraction was collected, centrifuged at 500 × g for 5 min, aliquoted, and stored at −80°C until needed (no longer than 3 weeks). The bacteria from the McLeod agar plates were harvested and suspended into PBS. Normalized bacteria stock solution of OD_600_ = 1.0 was used to make a working solution which contains 5 × 10^7^ bacteria in 1 ml of PBS. For each assay, 40 μl of bacteria working solution (containing 2 × 10^6^ bacteria) were added either to 160 μl of 100% serum (final concentration of 80%), or to 160 μl of 6.25% serum in PBS (final concentration of serum 5%), then the mixtures were incubated at 37°C for 90 min. One hundred and sixty microliters of PBS was used as a positive control (100% survival). Lysis was stopped by incubating the tubes on ice for 5 min. Surviving bacteria were enumerated by plating 10-fold serial dilutions of each suspension. Experiment was performed in a triplicate.

### Antibiotic susceptibility tests

For general tests with kanamycin, tetracycline (tetracycline hydrochloride, Zymo Research, Irvine, CA, USA), chloramphenicol, hygromycin B (Invitrogen, Carlsbad, CA, USA), gentamicin, polymyxin B sulfate, ampicillin, penicillin G (penicillin G sodium salt), and carbenicillin (Bioline, London, UK), we used gradient antibiotic plates (Szybalski and Bryson, 1952). For the determination of minimal inhibitory concentrations (MICs) for ampicillin, penicillin G, and carbenicillin broth macrodilution method was used.

### SDS-PAGE and western blot

*Francisella* strains expressing variants of DacD were cultured in Chamberlain medium (37°C, 200 rpm), and harvested by centrifugation after reaching OD_600_ of 1 (7,300 rpm, 10 min, 4°C). The cell pellets were washed with 50 mM Tris, pH 8 and resuspended in 50 mM Tris, pH 8 supplemented with protease inhibitor cocktail Complete Mini-EDTA free (Roche, Basel, Switzerland). Bacteria lysates were prepared using French press by three passages at 16,000 psi. Aliquots of lysates were separated on a one-dimensional SDS-PAGE and electroblotted onto PVDF membranes. Variants of DacD were detected by using a polyclononal rabbit anti FTS_1034 serum (Moravian—Biotechnology, Brno, Czech Republic). As secondary antibody the polyclonal swine anti-rabbit IgG/HRP (Dako, Santa Clara, CA, USA) was used. Chemiluminescence detection was employed by using a BM Chemiluminescence Blotting Substrate (POD) while following the manufacturer's instructions (Roche, Basel, Switzerland).

### Ethics statement

All experiments using mice were performed in accordance with guidelines of the Animal Care and Use Ethical Committee of the Faculty of Military Heath Sciences, University of Defense, Czech Republic. The research protocol was approved by this ethics committee under project no. 50-6/2016-684800. Experiments using human sera were conducted with the approval of Ethics Committee of University Hospital Hradec Kralove; reference no. 201710S10P and each volunteer provided written informed consent to participate in this study in accordance with regulatory guidelines.

### Statistical analysis

Statistical significances were analyzed by GraphPad Prism version 5 (GraphPad Software, La Jolla, CA, USA). The degree of significance was defined using two-way ANOVA followed by Bonferroni's multiple comparisons test or using Student's *t*-test depending on number of analyzed samples. In statistical analysis FSC200*/in dacD* mutant strain was compared to FSC200 strain. ^*^P < 0.05, ^**^P < 0.01, ^***^P < 0.001.

## Results

### Construction of mutant strain and functional complementation

To better characterize the role of *F. tularensis* DacD protein, we generated a FSC200 *dacD* insertional mutant (for details see Figure S1) introducing the retargeted mobile group II intron, as described previously (Rodriguez et al., 2008). To complement the mutant, we either expressed *dacD in trans* from GroES promoter of pKK289Km (Bönquist et al., 2008) or using the allelic exchange to return the gene *dacD* back on the chromosome by replacing gene with introduced intron (complementation *in cis*). Western blot analysis was used to confirm that the DacD protein is missing in the mutant strain and that the protein is produced in complemented strains (Figure S2).

### DacD role in intracellular replication, phagosomal escape, and cytopathogenicity

**Figure 1:**
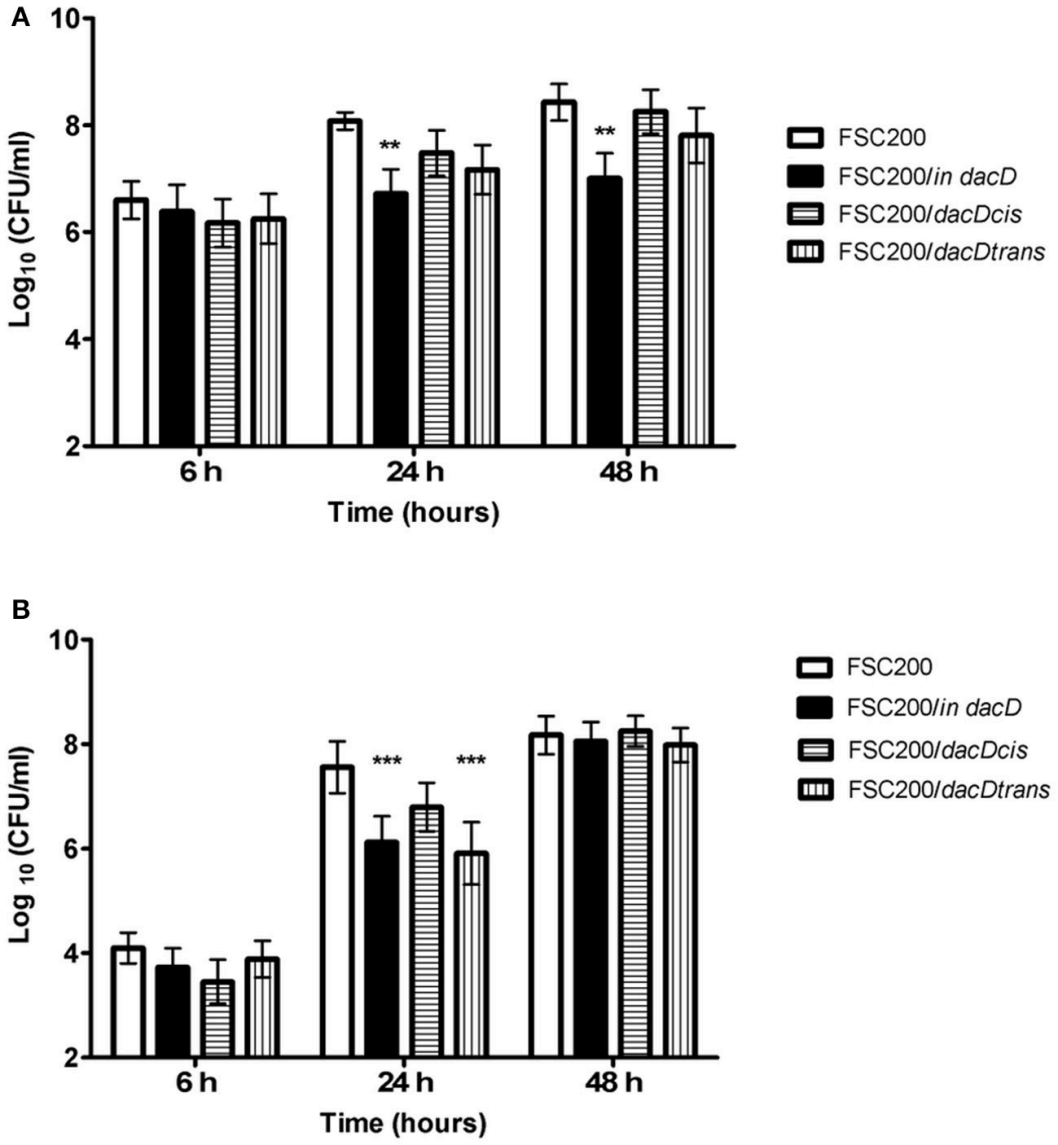
Intracellular growth of the FSC200*/in dacD* mutant strain is affected**. (A)** Replication inside bone-marrow derived macrophages. FSC200/*in dacD* mutant strain shows significantly reduced proliferation inside BMMs 24 and 48 h post infection. **(B)** Replication inside A549 cell line. The significant defect in replication in A549 cell line is visible only at time interval 24 h post infection. At 48 h post infection the replication rate of all strains is the same. **P < 0.01, ***P < 0.001.

To address the role of DacD in virulence of *F. tularensis* we first studied the intracellular replication of FTS200/*in dacD* mutant strain inside murine bone-marrow derived macrophages (BMMs) and type II pulmonary epithelial A549 cell line. At 24 h post infection, the replication defects were obvious for both these types of cells. When compared to the wild-type FSC200 strain, the mutant bacteria showed significantly lower numbers of bacteria/ml (Figure 1). This defect was eliminated by complementation *in cis* but not by complementation *in trans*. The different situation has occurred 48 h after infection. It is evident that the defect in replication is still maintained in BMMs, whereas in A549 the FSC200/*in dacD* mutant strain proliferates comparable to WT. These results demonstrate the differences in requirement of DacD for intracellular proliferation and survival in BMMs and A549 cell line.

**Figure 2:**
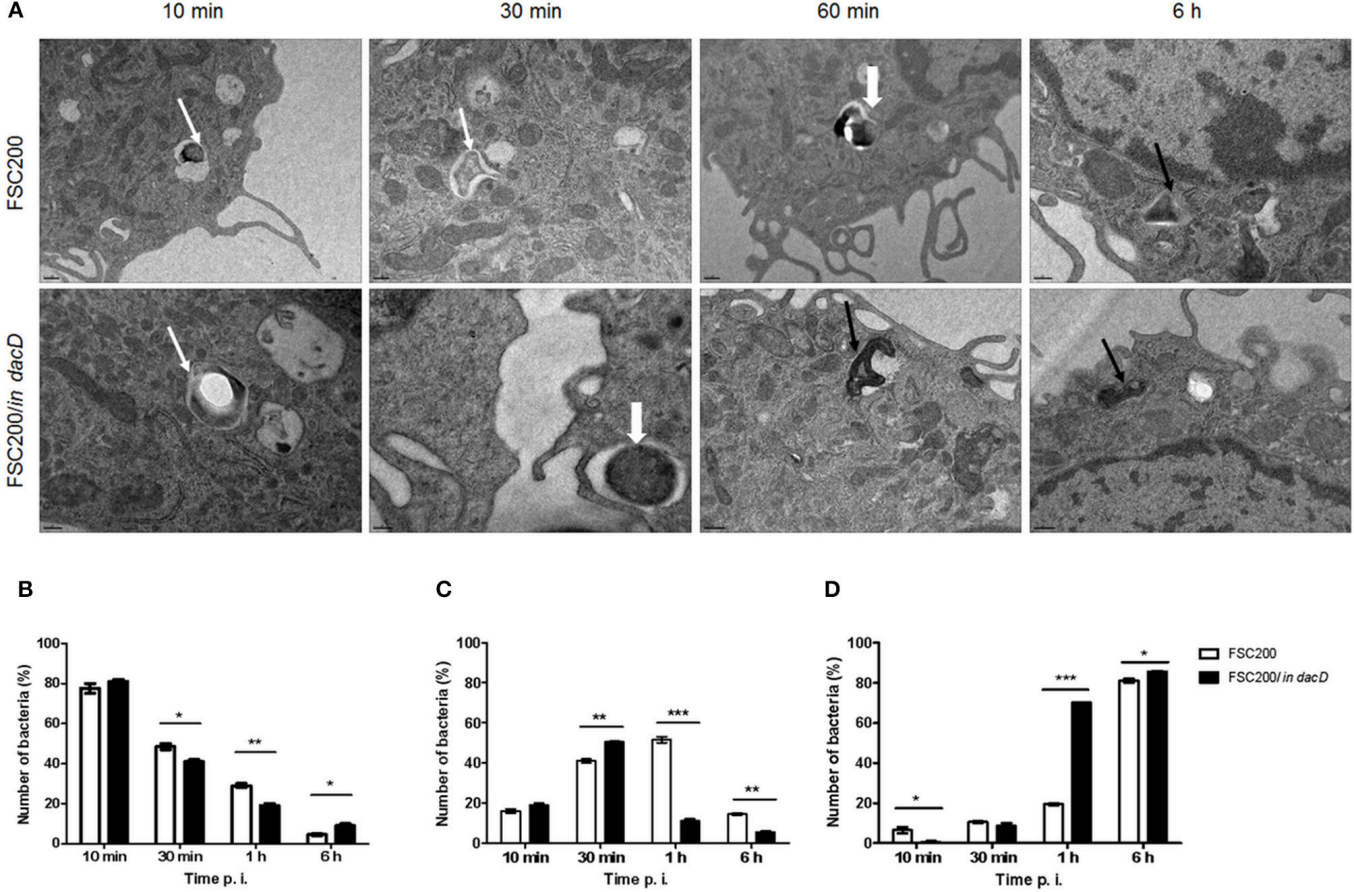
Intracellular fate of FSC200/*in dacD*. **(A)** Representative transmission electron microscopy images of BMMs infected with wild-type and FSC200/*in dacD* mutant strains at indicated time intervals after infection. Thin white arrows show intact phagosome, thick white arrows show damaged phagosomal membrane, while thin black arrows show bacteria free in the cytosol. **(B–D)** Quantification of bacteria inside intact phagosomes **(B)**, phagosomes with damaged membrane **(C)**, and bacteria free in cytosol **(D)**. *P < 0.05, **P < 0.01, ***P < 0.001.

Transmission electron microscopy was used to further analyze the intracellular fate of the FSC200/*in dacD* mutant bacteria. The BMMs were infected by the wild-type and mutant bacteria, respectively. In four time intervals of infection, the percentage of bacteria in intact phagosome, damaged phagosome and in cell cytosol was calculated (Figure 2). Interestingly, we found out that mutant bacteria escape the phagosomes more rapidly than wild-type bacteria. The most significant differences were observed 1 h post infection. At this time only 11% of the mutant bacteria resided in damaged phagosomes vs. 51.5% of the wild-type, and 70 vs. 19.5% are found to be free in cytosol, respectively. This finding suggests that the inactivation of DacD impaired intracellular replication of mutant bacteria but it did not affect phagosomal escape. These data were confirmed by the analysis of colocalization of FSC200/*in dacD* bacteria- containing phagosomes with EEA1, LAMP1, and cathepsin D (data not shown).

To investigate the role of the DacD protein on *Francisella* cytopathogenic effects we infected BMMs with the wild-type FSC200 strain or the FSC200/*in dacD* mutant and measured the release of lactate dehydrogenase (LDH) into the cell supernatant. At the beginning of infection (time 0 h), the LDH level released from cells infected with the FSC200/*in dacD* mutant (~17%) was comparable to the LDH release detected in cells infected with the wild-type FSC200 strain (~15%; Figure S3). After 24 h of infection, the LDH levels increased to 49 and 38% for the FSC200/*in dacD* and parental FSC200 strains, respectively. At 48 h postinfection, the level of LDH release was 56% for the FSC200/*in dacD* mutant strain, which was almost the same as the LDH release detected for the wild-type FSC200 strain (53%). The LDH assay showed that the FSC200/*in dacD* mutant strain induces loss of host cell membrane integrity at similar levels as the wild-type strain.

### Animal studies

**Figure 3:**
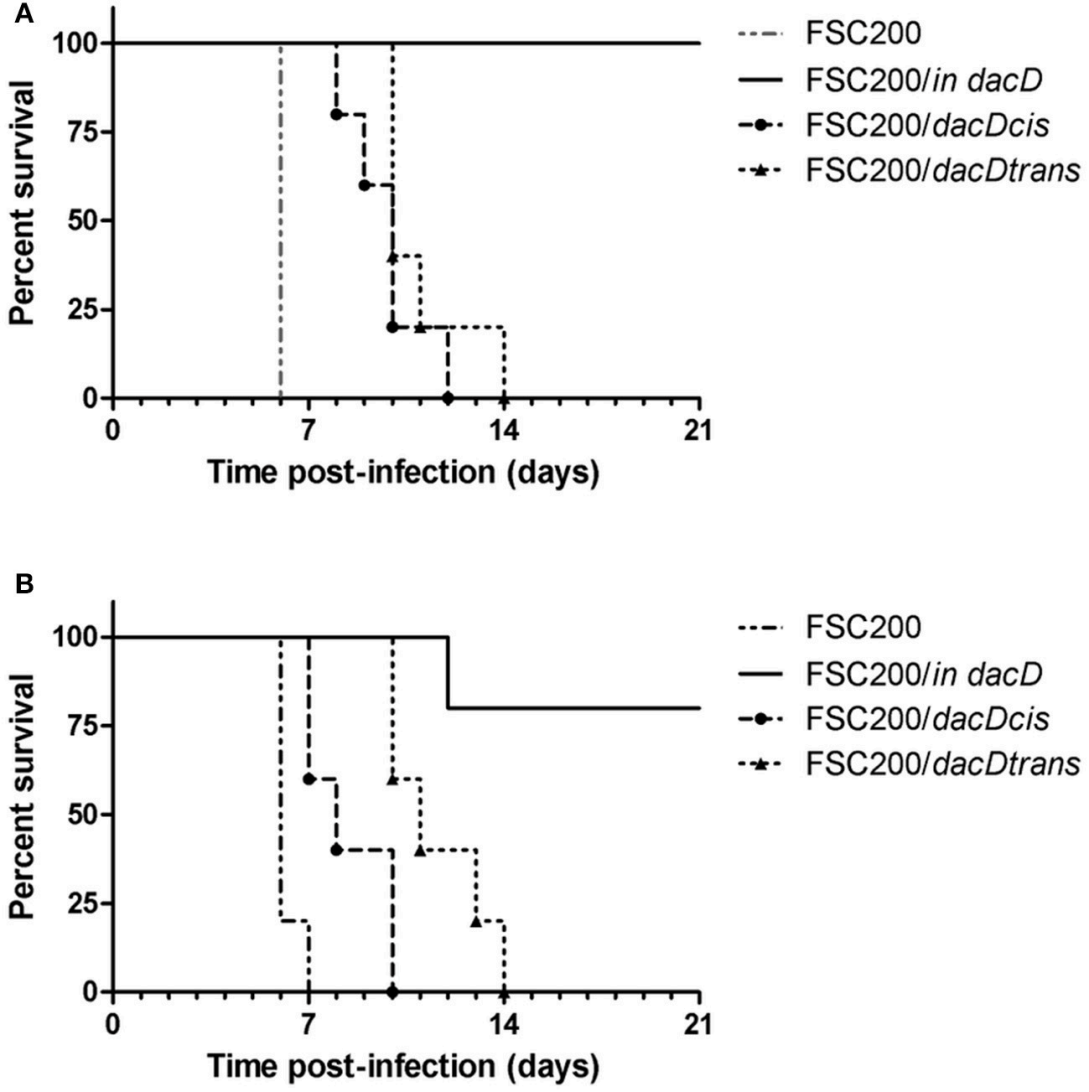
Survival of mice after subcutaneous infection with FSC200/*in dacD*, complemented and wild-type strains. Mice were infected with a dose of 10 cfu/mouse **(A)** and 50 cfu/mouse **(B)** and were observed for 21 days and the deaths were recorded.

Taken into account that the mutant strain is deficient in intracellular replication, we further verified its attenuation in mouse model of infection. The course of mouse infection was followed for 21 days. Using the dose of 10^2^ cfu of FSC200/*in dacD* resulted in the death of all mice tested (data not shown). By sequential dose reduction, we found out that the mutant strain is less lethal than wild-type strain for mice challenged by subcutaneous route at dose of 10 CFU/mouse (Figure 3A). The increase of the mutant strain dose to 50 cfu led to the death of one animal (Figure 3B). So it is evident that opposite the wild-type strain, the FSC200/*in dacD* mutant is *in vivo* attenuated but it is not avirulent. The complementation *in cis* and *in trans* restored the wild-type phenotype with 6–8 days delay for the dose of 10 cfu and 3–7 days delay for the dose of 50 cfu when compared to the original wild-type strain.

**Figure 4:**
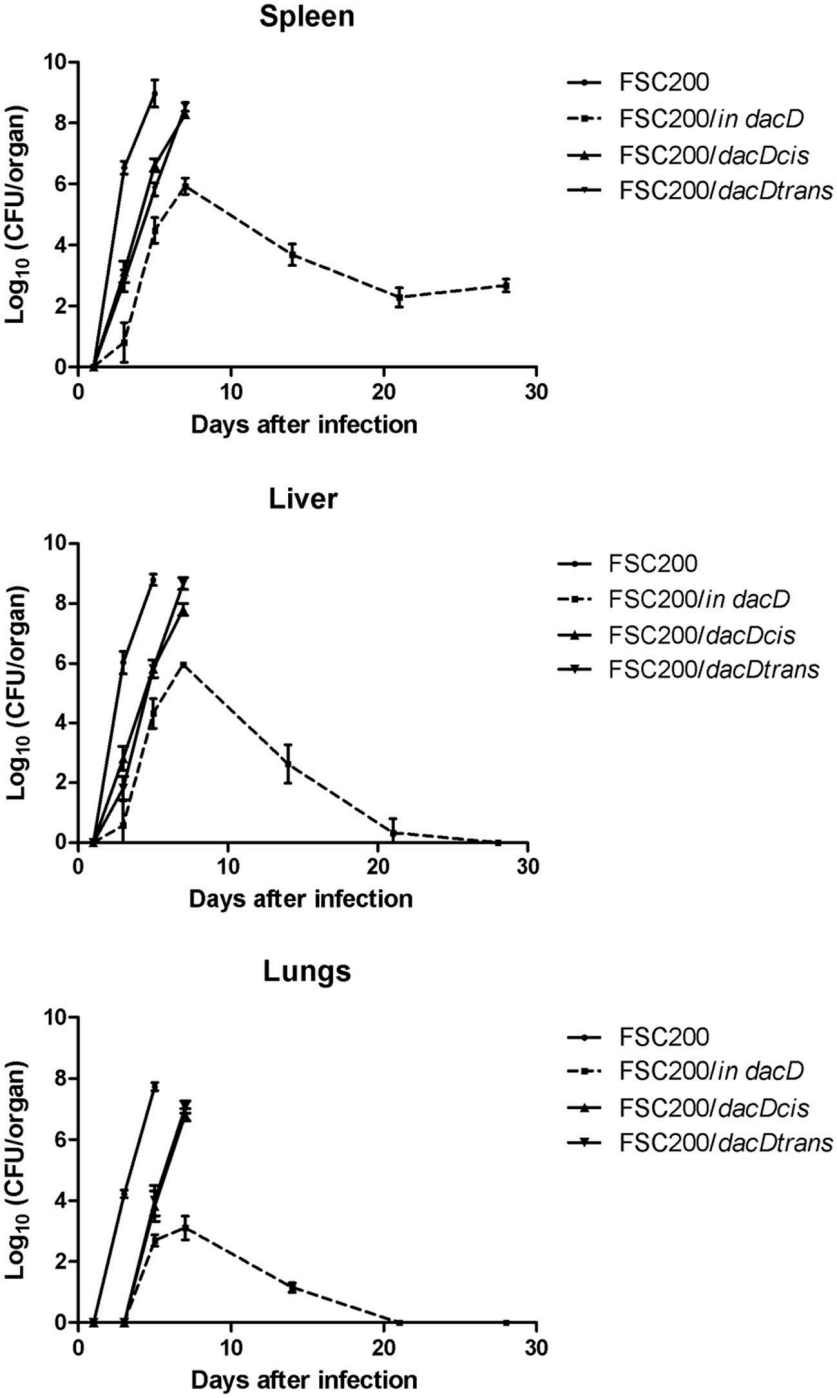
Kinetics of FSC200/*in dacD* replication inside target host organs after s.c. infection. The groups of 3 BALB/c mice were infected s.c. with 10 CFU of the wild-type and mutant strains of *F. tularensis*. At the given time points, the numbers of bacteria in target organs were enumerated by CFU counts.

The mutant attenuation was further corroborated by the analysis of bacteria dissemination in host tissues. The groups of three mice were challenged by subcutaneous route at the dose of 10 cfu/mouse for each tested strain. We followed the kinetics of infection by assessing the numbers of viable bacteria in the spleen, liver, and lung tissues at the post-infection time points. None of the mice infected with the wild-type FSC200 strain survived more than 5 days post infection, and mice infected with complemented strains dying gradually between day 7 and 14 post infection due to the rapid progression of disease. Contrary, mice infected with FSC200/*in dacD* survived the infection and the bacterial loads in target organs did not reach those for wild-type and complemented strains (Figure 4). For s.c. infection, the maximum replication of mutant strain inside tissues were detected on day 7. The FSC200/*in dacD* strain reached ~10^6^ cfu/organ in case of spleen and liver but 10^4^ cfu/organ in the lungs. After day 7, the number of mutant bacteria slowly decline, at day 21 mutant were totally cleared out of lung and for liver at day 28. However, mutant bacteria were not totally cleared out of spleen during whole observed time period. These results indicate that the FSC200*/in dacD* mutant is able to infect mice and persist in organs, but replicates less effectively than wild-type strain inside the host tissues.

### Sensitivity of the FSC200/*in dacD* to stress stimuli

**Figure 5:**
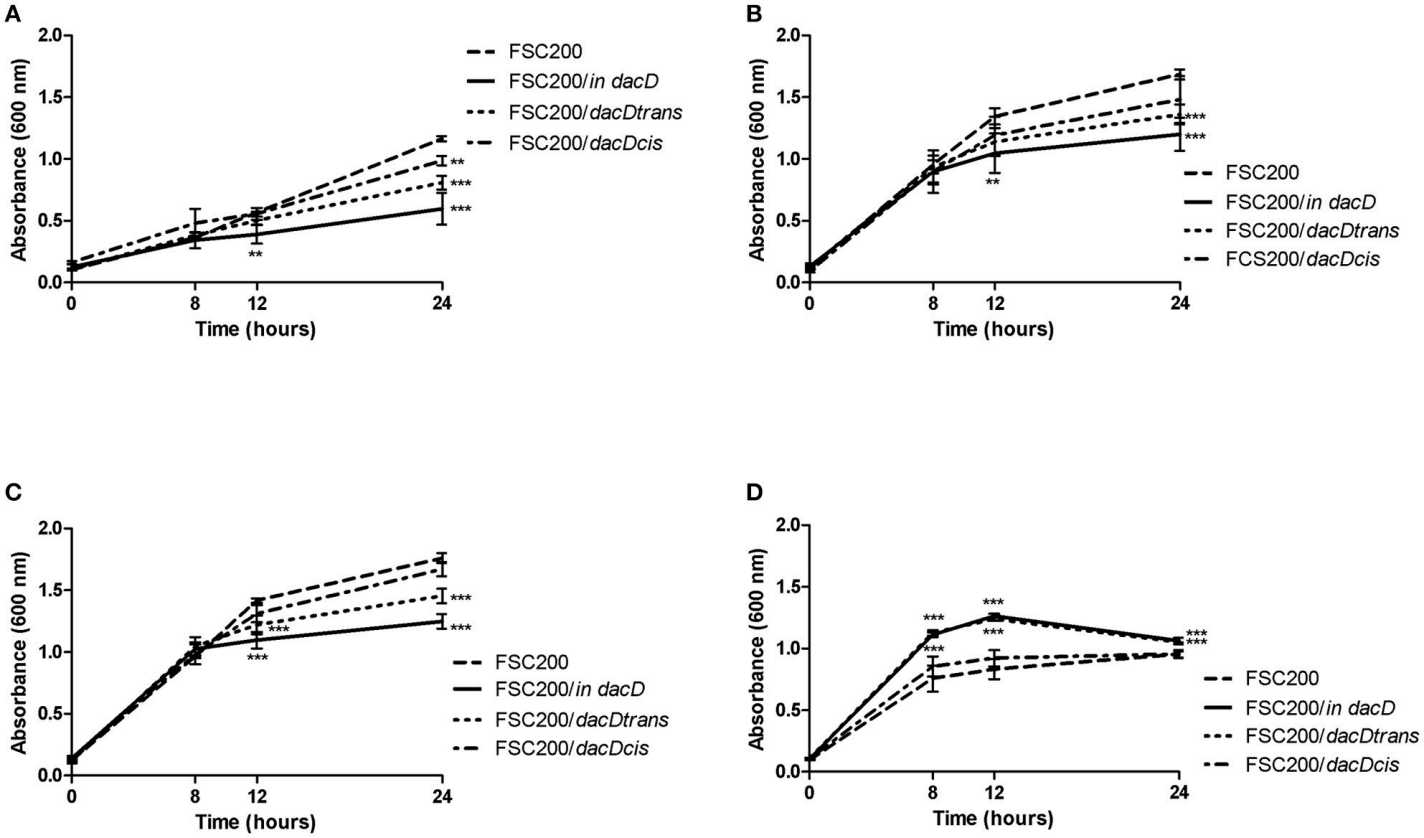
Stress growth kinetics. *F. tularensis* strains were cultivated in altered Chamberlain medium (CMH) to simulate various stress conditions and the growth kinetics was determined by measuring the OD_600_ using microplate reader FLUOstar Optima (BMG Labtech) for 24 h. **(A)** Growth in CHM supplemented with 2% NaCl. **(B)** Growth in elevated temperature (42°C). **(C)** Growth in medium with pH 4. **(D)** Growth in presence of 20 μM CuCl_2_. **P < 0.01, ***P < 0.001.

**Figure 6:**
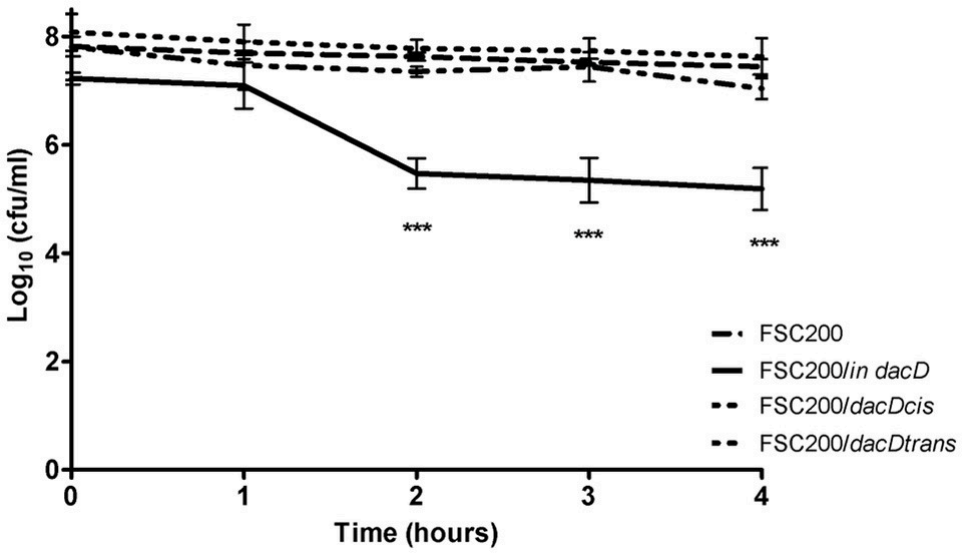
SDS survival assay. *F. tularensis* strains were cultivated in CHM and when the strains reached the OD_600_ = 1, the SDS was added and the numbers of CFU were enumerated at indicated time intervals. ***P < 0.001.

To address the role of DacD protein in adaptation to various stress stimuli, we compared the growth rate between wild-type strain and all the mutant strains. All tested strains grew in the media with four conditions as following: acid pH (pH 4), in high osmolarity (3% NaCl), in 42°C or CuCl_2_-induced oxidative stress (Figure 5). As was expected the mutant strain was not able to resist high osmolarity, high temperature and low pH (Figures 5A–C). On the other hand the FSC200/*in dacD* mutant strain grew better than wild-type under the oxidative stress conditions induced by the presence of 20 μM CuCl_2_ with the maximum of growth at 12 h interval, but afterwards the growth slowly drop down (Figure 5D). At standard cultivation conditions, the growth kinetics of wild-type and FSC200/*in dacD* mutant strain did not significantly differ (data not shown). With the exception of high osmolarity the complementation *in cis* restored fully wild-type phenotype, whereas the complementation *in trans* did not. Further, we studied the SDS sensitivity of the mutant strain. At 1 h after SDS addition no difference in bacterial growth was seen, but in later time intervals (2, 3, and 4 h) the significant difference in CFU count was observed (Figure 6). This result clearly indicates the membrane defects in FSC200/*in dacD* bacteria. Both complemented strains as well as wild-type strain resist the SDS during whole time interval tested.

### FSC200/*in dacD* shows morphological changes and membrane defects

**Figure 7:**
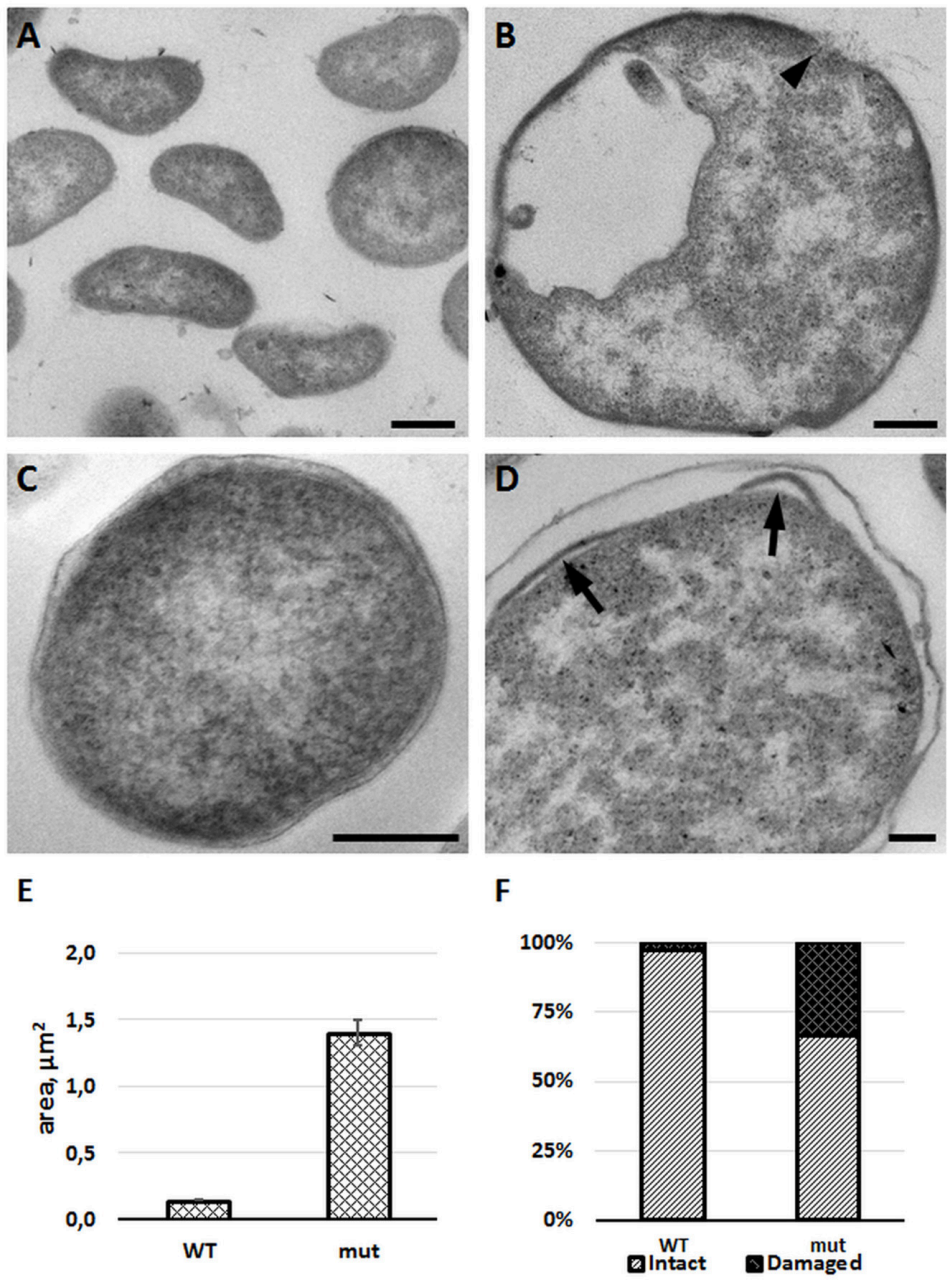
FSC200/*in dacD* mutant bacteria differ in size when compared to the wild-type and exhibit membrane defects. *F. tularensis* FSC200 wild-type strain **(A,C)** and FSC200/*in dacD* mutant strain **(B,D)** were cultivated in CHM and then processed for transmission electron microscopy. Typical electron micrographs of ultrathin sections of bacteria in **(A,B)** at the same magnification illustrate size difference between wild-type and mutant strains. Wild-type cells possess intact membrane **(C)**, while mutant cells exhibit discontinuous membrane (**B**, arrowhead) or membrane bulging (**D**, arrows). **(E)** Graph showing mean cell profiles area of wild-type and mutant bacteria on ultrathin sections; **(F)** Quantification of incidence of cell profiles with membrane defects on ultrathin sections. Bars: **(A,B)** 200 nm; **(C,D)** 100 nm.

To confirm membrane defects, we studied the morphology of mutant bacteria by using transmission electron microscopy. Comparing to the wild-type strain, the FSC200/*in dacD* bacteria exerted larger size and discontinuous plasma membrane, as well (Figure 7). Morphological observation was supported by quantification. Mean area of cell profiles on ultrathin sections of mutant cells was nearly 10 times higher than in wild-type strain. Number of profiles membrane defects were visible on every third profile in mutant cells sections. Taken into account that the 80-nm thick section represents only a small fraction of the total cell volume, the total incidence of membrane defects in cell population would be much higher. These results demonstrate the necessity of DacD protein for the maintaining of bacterial membrane integrity and bacterial shape.

### FSC200/*in dacD* mutant strain sensitivity to bactericidal effect of human serum

**Figure 8:**
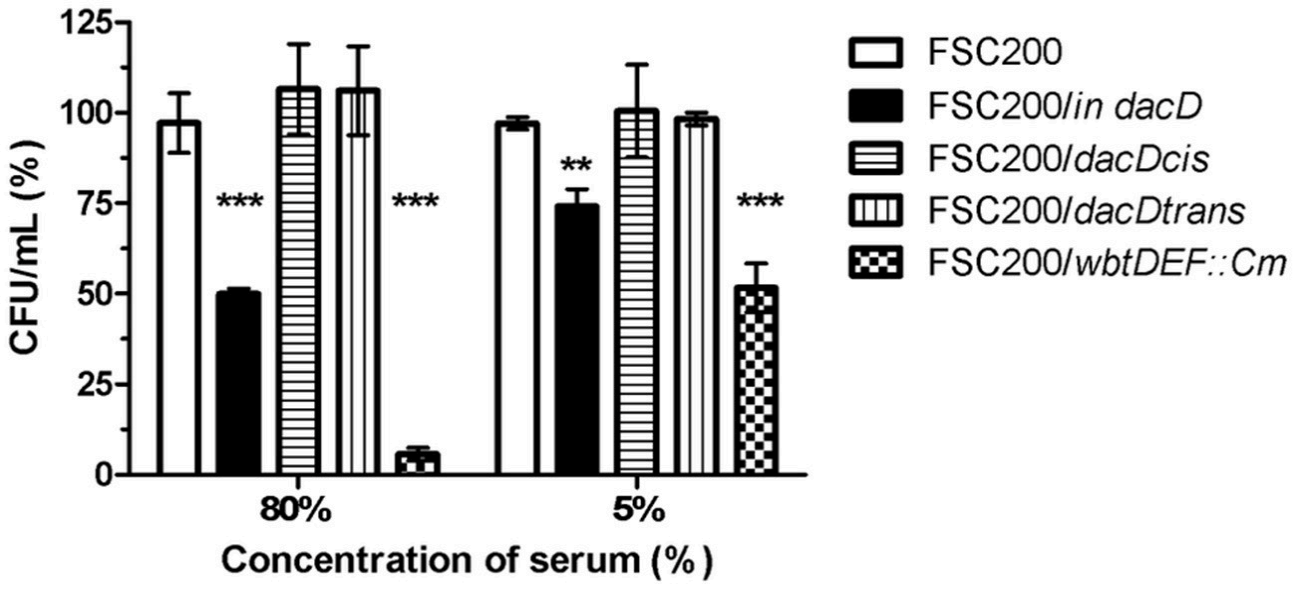
Sensitivity to bactericidal effects of human serum. FSC200, FSC200/*in dacD*, FSC200/*dacDtrans*, FSC200/*dacDcis* and FSC200/*wbtDEF::Cm* strains were incubated with human nonimmune serum and their resistance/susceptibility was determined by CFU counts. FSC200/*wbtDEF::Cm* strain is highly susceptible to the effects of complement and the strain was used as a positive control for serum complement activity. **P < 0.01, ***P < 0.001.

The membrane defects can influence bacterial resistance to human serum. Therefore, we performed the serum bactericidal assay in which the wild-type, FSC200/*in dacD* mutant, both complemented mutant strains, and the FSC200/*wbtDEF::Cm* strain lacking LPS, were exposed to human nonimmune serum. The significant sensitivity of FSC200/*wbtDEF::Cm* strain to human serum on the one hand and the resistance of FSC200 on the other hand were consistent with previously published data (Dankova et al., 2016). The FSC200/*in dacD* mutant strain also exhibited some degree of sensitivity to complement-mediated lysis. In the case of 5% human serum 25% of mutant bacteria were killed while using 80% human serum only 50% of bacteria survived (Figure 8). Both complemented strains showed resistance to effects of serum comparable to wild-type level.

### Susceptibility to antibiotics

Because the surface structures of FSC200/*in dacD* showed slight defects, the question arose as whether or not the mutant strain might be more sensitive to antibiotics, especially β-lactams. Using gradient antibiotic plates we tested susceptibility of FSC200, both complemented strains and FSC200/*in dacD* to kanamycin, tetracycline, chloramphenicol, hygromycin B, gentamicin, polymyxin B sulfate, ampicillin, penicillin G, and carbenicillin. From this experiment it seemed the resistance to β-lactams might be influenced because growth of the mutant strain was not as convincing as the other strains (data not shown). The susceptibility to tetracycline, chloramphenicol, hygromycin B, gentamicin, polymyxin B sulfate was not affected and was the same as for wild type and complemented strains. In case of kanamycin as expected the strain complemented *in trans* carrying the plasmid with a kanamycin cassette showed higher resistance to it. In order to check if there are differences in sensitivity to β-lactams, the broth macrodilution method for determination of MIC values was adopted. Nevertheless, in the concentration range tested (0–1,000 μg/ml) no differences were detected.

## Discussion

Bacterial cell wall and the peptidoglycan layer are necessary to maintain cell shape and prevent cell lysis. To synthesize or modify the peptidoglycan cell wall bacteria possess a machinery of enzymes. A small group of them forms PBPs. These enzymes catalyze known biochemical reactions but the physiological role of the LMW PBPs still remains unclear (Ghosh et al., 2008). Here we aimed to address the role of DD-carboxypeptidase DacD, one of the LMW PBPs, in *Francisella*. This protein was identified as one of the proteins with altered abundance in membrane enriched fraction of *dsbA* deletion mutant strain compared to its wild counterpart (Pavkova et al., 2017). DsbA is believed to ensure the correct folding of many proteins, mainly virulence factors through its oxidoreductase and isomerase activities and thus DsbA indirectly promotes host cell binding, invasion, intracellular survival, or other virulence functions (Rowe and Huntley, 2015). As expected, we showed that the inactivation of DacD does not influence the viability of the mutant bacteria confirming the dispensability of this protein not only in *E. coli, S. enterica*, and *Streptomyces coelicolor*, but also in *F. tularensis* (Brambilla et al., 2014; Rioseras et al., 2016). On the other hand we proved that the virulence of *Francisella* is affected by insertion inactivation of *dacD* gene. The mutant strain FSC200/*in dacD* showed defects in intracellular replication which was more pronounced in bone marrow derived macrophages. Previously, it was proposed in two comprehensive studies by Asare (Asare and Abu Kwaik, 2010; Asare et al., 2010) that a transposon insertion mutant in the DacD homolog gene in *F. novicida* has decreased proliferation within *Drosophila melanogaster*-derived S2 cells. This phenomenon might be connected either with the replication defect or inability to escape the phagosome. TEM analysis revealed that in case of FSC200/*in dacD* mutant the phagosomal escape was not diminished. On the contrary, the mutant bacteria escaped the phagosome faster than wild-type bacteria but failed to replicate with the same kinetics as the wild-type strain. The same effect was observed for example in *F. novicida iglD* mutant (Santic et al., 2007). The authors assumed that the mutant was probably unable to modify the cytosol of macrophages to render it permissive to bacterial replication. Here, we speculate that the decreased resistance to acid environment might be the reason for rapid phagosomal escape of the FSC200/*in dacD* mutant. Noteworthy, there are discrepancies concerning phagosome acidification and some authors claim that *F. tularensis* does not require acidification of phagosome for the escape (Clemens et al., 2009).

So far only a few genes have been identified as being specifically required for cytosolic replication by *Francisella* (Brotcke et al., 2006; Fuller et al., 2008; Pechous et al., 2008; Alkhuder et al., 2009; Wehrly et al., 2009; Chong et al., 2012). Interestingly, mutants in all of these genes have also been strongly attenuated *in vivo*. This is not case of the FSC200/*in dacD* mutant that exhibits residual virulence. Nevertheless, the assessment of the viable mutant bacteria in target organs proved their lower numbers and gradual elimination with exception for spleen where survived during whole observed time interval.

The lack of functional DacD is also reflected by increased sensitivity to the bactericidal serum effect. The resistance of *Francisella* to this phenomenon has been attributed to LPS, so far (Sorokin et al., 1996). Thus, the explanation might be (i) that this effect is indirect or (ii) DacD might be involved in LPS biosynthesis, as well, but no evidence collected thus far have been able to support these hypotheses.

Recently it has been described that *E. coli* DacD is important for cell shape maintenance in acidic growth medium (Peters et al., 2016). The data we collected were in agreement as observations documented the mutant strain being unable to resist the acid pH as effectively as the wild-type strain and grew slowly in media with acid pH (pH 4). Similarly, the mutant bacteria had exerted the increased sensitivity to high temperature and high osmolarity. Nevertheless, these growth defects were not fully compensated by complementation *in trans*. Additionally, we demonstrate that the disruption of *dacD* gene results in insufficient resistance to surface active reagent SDS. This susceptibility as indicated by TEM analysis described membrane bulging. The discontinuous membrane may be associated with the defects in bacterial membrane integrity. The TEM quantification revealed the membrane defects may have over a third of the mutant population. The size of mutant bacteria were also altered, bacteria were almost 10 times larger when compared to the wild-type, which could suggest certain disproportion in peptidoglycan and outer membrane synthesis.

Assuming that the phenotype changes in the mutant strain might be a result of the polar effect on the genes flanking *dacD*, we have searched for alterations in their transcription. All monitored genes FTS_1038 through FTS_1032 are transcribed in the mutant, but the transcription level of FTS_1033 and FTS_1032 is lower than that in the wild-type (data not shown). In strain complemented *in cis* the transcription levels were those as for wild-type strain. However, the decreased transcription level of the FTS_1033 and FTS_1032 genes has also been observed in the strain complemented *in trans* with fully restored phenotype only in part of the tested assays. Therefore, we could assume that the changes in intramacrophage replication, attenuated phenotype in mouse model and the membrane defects are results of solely inactivation of *dacD* (complementation *in trans* restores the wild-type phenotype), but differences of mutant strain in resistance to acidic pH, high temperature and high osmolarity might be also the consequence of decreased transcription of downstream genes in the mutant (complementation *in trans* does not fully restore the wild-type phenotype).

As the DacD is enzyme connected with peptidoglycan layer modulation the higher susceptibility to especially β-lactams was considered. But in accordance with Sarkar et al. (2011) the inactivation of DacD in *Francisella* did not change the β-lactam resistance similarly as was shown for *E. coli*. Furthermore, it is known that *F. tularensis* codes for functional β-lactamase, AmpG and metallo-β-lactamase family protein which hydrolyze β-lactam antibiotics (Biswas et al., 2008).

In conclusion, our results provide the evidence that the DacD protein has a role in *Francisella* pathogenesis. This is in agreement with previously published data where the expression of gene coding for FTT_1029 protein (DacD homolog in *F. tularensis* subsp. *tularensis* SchuS4 strain) was found to be up-regulated during intramacrophage growth (Wehrly et al., 2009) and where the FTT_1029 protein was described as virulence factor candidate (Wallqvist et al., 2015). In several recent publications DacD homologs were also identified as substrates for protein DsbA in *F. tularensis* subsp. *holarctica* LVS, *F. tularensis* subsp. *tularensis* SchuS4 and *F. tularensis* subsp. *holarctica* FSC200 (Ren et al., 2014; Qin et al., 2016; Pavkova et al., 2017; Spidlova et al., 2017) or as interacting partner of another virulence factor, TPR1 protein (FTL_0205) in *F. tularensis* subsp. *holarctica* LVS (Dieppedale et al., 2013).

## Author contributions

PeS, MS, and JS conceived and designed the experiments. PeS, PaS, VD, IS, MS, DP, and VP performed the experiments. PeS, PaS, MS, DP, VP, and JS analyzed the data. PeS, PaS, MS, VP, and JS wrote the paper. All authors reviewed and approved the manuscript.

### Conflict of interest statement

The authors declare that the research was conducted in the absence of any commercial or financial relationships that could be construed as a potential conflict of interest. The reviewer MM declared his involvement as a co-editor in the Research Topic with one of the authors MS, and confirmed the absence of any other collaboration.
