# Supplementary material for: Francisella tularensis D-Ala D-Ala Carboxypeptidase DacD Is Involved in Intracellular Replication and It Is Necessary for Bacterial Cell Wall Integrity
Source: Front Cell Infect Microbiol. 2018 Apr 10;8:111. doi: 10.3389/fcimb.2018.00111 (PMC5903032; doi:10.3389/fcimb.2018.00111)
Supplement: Supplementary file 2 [file Image2.pdf]

Supplementary Material

Francisella tularensis D-Ala D-Ala carboxypeptidase DacD is involved in intracellular replication and it is necessary for bacterial cell wall integrity

*Petra Spidlova[1], Pavla Stojkova[1], Vera Dankova[1], Iva Senitkova[1], Marina Santic[2], Dominik Pinkas[3], Vlada Philimonenko[3,4], and Jiri Stulik[1]
*Correspondence: Petra Spidlova, petra.spidlova@unob.cz

1. Supplementary Figures

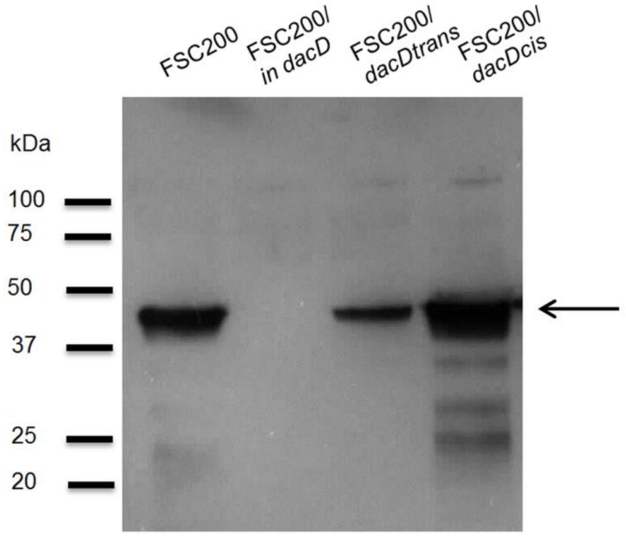

Supplementary Figure 2. Immunodetection of DacD protein in cell lysates of *F. tularensis* wild-type strain, FSC200/*in dacD* mutant strain, and strains complemented *in trans* and *in cis*. The positions of precision plus protein kaleidoscope prestained standards (Bio-Rad) are indicated on the left (in kDa). The position of DacD protein is indicated by black arrow.
