# Supplementary material for: Francisella tularensis D-Ala D-Ala Carboxypeptidase DacD Is Involved in Intracellular Replication and It Is Necessary for Bacterial Cell Wall Integrity
Source: Front Cell Infect Microbiol. 2018 Apr 10;8:111. doi: 10.3389/fcimb.2018.00111 (PMC5903032; doi:10.3389/fcimb.2018.00111)
Supplement: Supplementary file 4 [file Table1.PDF]

Supplementary Material

Francisella tularensis D-Ala D-Ala carboxypeptidase DacD is involved in intracellular replication and it is necessary for bacterial cell wall integrity

*Petra Spidlova[1], Pavla Stojkova[1], Vera Dankova[1], Iva Senitkova[1], Marina Santic[2], Dominik Pinkas[3], Vlada Philimonenko[3,4], and Jiri Stulik[1]
*Correspondence: Petra Spidlova, petra.spidlova@unob.cz

2. Suplementary Table

Table S1: Primers used in this study.

Primer	Sequence	Application
DacD_IBS	5´AAAACTCGAGATAATTATCCTTACTCGCCCAG AAAGTGCGCCCAGATAGGGTG-3´	Targetron mutagenesis
DacD_EBS1	5´CAGATTGTACAAATGTGGTGATAACAGATAAG TCCAGAAAAATAACTTACCTTTCTTTGT-3´	
DacD_EBS2	5´TGAACGCAAGTTTCTAATTTCGGTTGCGAGTC GATAGAGGAAAGTGTCT-3´	
DacD_R	5´CATTACCAGACACTACATCCATAC-3´	
DacD_F	5´GCGGGGGTAGCTTTTG-3´	
DacD_pKK_F	5´CATATGAAATTGACAAAAATAGCTTT-3´	complementation *in trans*
DacD_pKK_R	5´GAATTCTTACCACCAACCTTTTTGTG-3´	
DacDcis_F	5´TCTAGAGTACAAAAGCTCCTCCACCAAA-3´	complementation *in cis*
DacDcis_R	5´CTCGAGGTACATTCGCAAATAAGGAGTAT-3´	
DacDcis_S1	5´TATTTATTGAGTTTCTTGAGTGA-3	sequencing
DacDcis_S2	5´ACAAAGAGTCTATCACAAAAAC-3´	
DacDcis_S3	5´ATCGTAAACAGTGCTTACCTAA-3´	
DacDcis_S4	5´GAAAGTAATAATCACAATCAACAA-3´	
DacDcis_S5	5´ĆTAAAGGCTCATATTATTGATG-3´	
